# The involvement of RIPK4 in TNF-α-stimulated IL-6 and IL-8 production by melanoma cells

**DOI:** 10.1007/s00432-024-05732-3

**Published:** 2024-04-24

**Authors:** Ewelina Madej, Anna Lisek, Anna A. Brożyna, Agnieszka Cierniak, Norbert Wronski, Milena Deptula, Anna Wardowska, Agnieszka Wolnicka-Glubisz

**Affiliations:** 1 Department of Biophysics and Cancer Biology, Faculty of Biochemistry, Biophysics and Biotechnology, Jagiellonian University, Gronostajowa 7, 30-387 Kraków, Poland; 2 Department of Human Biology, Insitute of Biology, Faculty of Biological and Veterinary Sciences, Nicolaus Copernicus University, Lwowska1, 87-100 Toruń, Poland; 3 Department of Biochemistry, Faculty of Medicine and Health Sciences, Andrzej Frycz Modrzewski Krakow University, Kraków, Poland; 4 Doctoral School of Exact and Natural Sciences, Jagiellonian University, Kraków, Poland; 5 Laboratory of Tissue Engineering and Regenerative Medicine, Division of Embryology, Faculty of Medicine, Medical University of Gdańsk, Gdańsk, Poland; 6 Department of Physiopathology, Faculty of Medicine, Medical University of Gdańsk, Gdańsk, Poland

**Keywords:** RNA-seq, RIPK4, Melanoma, Cytokines, BIRC3, Transcriptome, p-38

## Abstract

**Purpose:**

The receptor-interacting protein kinase (RIPK4) has an oncogenic function in melanoma, regulates NF-κB and Wnt/β-catenin pathways, and is sensitive to the BRAF inhibitors: vemurafenib and dabrafenib which lead to its decreased level. As its role in melanoma remains not fully understood, we examined the effects of its downregulation on the transcriptomic profile of melanoma.

**Methods:**

Applying RNA-seq, we revealed global alterations in the transcriptome of WM266.4 cells with RIPK4 silencing. Functional partners of RIPK4 were evaluated using STRING and GeneMANIA databases. Cells with transient knockdown (via siRNA) and stable knockout (via CRISPR/Cas9) of RIPK4 were stimulated with TNF-α. The expression levels of selected proteins were assessed using Western blot, ELISA, and qPCR.

**Results:**

Global analysis of gene expression changes indicates a complex role for RIPK4 in regulating adhesion, migration, proliferation, and inflammatory processes in melanoma cells. Our study highlights potential functional partners of RIPK4 such as BIRC3, TNF-α receptors, and MAP2K6. Data from RIPK4 knockout cells suggest a putative role for RIPK4 in modulating TNF-α-induced production of IL-8 and IL-6 through two distinct signaling pathways—BIRC3/NF-κB and p38/MAPK. Furthermore, increased serum TNF-α levels and the correlation of RIPK4 with NF-κB were revealed in melanoma patients.

**Conclusion:**

These data reveal a complex role for RIPK4 in regulating the immune signaling network in melanoma cells and suggest that this kinase may represent an alternative target for melanoma-targeted adjuvant therapy.

**Supplementary Information:**

The online version contains supplementary material available at 10.1007/s00432-024-05732-3.

## Introduction

The receptor-interacting serine/threonine kinase 4 (RIPK4) is a member of the RIP family whose significant role in regulating a variety of host defense functions has been demonstrated, including control of inflammatory gene expression, various forms of cell death, and maintaining cutaneous and intestinal barrier functions (Cuny and Degterev 2021). The catalytic activity of RIPK4 has been shown to play a crucial role in normal epidermal differentiation in mice and in maintaining skin barrier function (De Groote et al. 2015; Kwa et al. 2014; Oberbeck et al. 2019; Xu et al. 2020). Currently, numerous studies are underway to comprehend the functional role of RIPK4 kinase in cancer initiation and progression. Several studies indicate that RIPK4 can act as both a tumor suppressor and a tumor oncogene, depending on the type of cancer (Xu et al. 2020). While it has been reported that RIPK4 is involved in skin tumorigenesis by regulating DLV2 or Pkp1 phosphorylation (Huang et al. 2013; Lee et al. 2017), our recent study shows an important role of this kinase in melanoma progression by involvement in NFκB and Wnt/β-catenin transduction signaling leading to increased migration, invasive potential, and cell proliferation as well tumor growth in vivo (Madej et al. 2021; Wronski et al. 2024).

Melanoma, characterized by an increasing incidence rate and high mortality once metastasis occurs, arises from transformed melanocytes in the skin, eye, or mucosa, contributing to over 75% of skin tumor-related deaths (Gershenwald and Guy 2016). The poor prognosis is related to the low susceptibility of metastatic melanoma (MM) to chemotherapy, resulting in a 5-year survival rate of only 15% (Pacheco et al. 2011). Recent advances in clinical approaches, such as the combined inhibition of BRAF^V600^ and MEK and of immune checkpoint therapy for advanced-stage melanomas, have significantly reduced melanoma mortality (Bai and Flaherty 2021; Sacchetto et al. 2021). However, despite these advancements, morbidity and mortality remain high, as a substantial proportion of patients develop primary or secondary resistance to immunotherapy and/or BRAF/MEK inhibitors (Hawthorne et al. 2020; Siegel et al. 2020; Arozarena and Wellbrock 2019; Gide et al. 2018). Melanoma is recognized for its ability to produce immunogenic neoantigens, making it one of the most immunogenic tumors. The melanoma antigens include both common antigens on melanoma cells and various normal cells, differentiation antigens on melanoma cells, melanocytes and their precursors, and specific antigens on melanoma cells. The exact mechanism of the antigen expression and its presentation is still unknown, but its relationship with immune cells resident in the microenvironment probably influences cancer cell proliferation, progression, and metastasis. The clinical expression of melanoma immunogenicity is quite frequent relative spontaneous remission, the presence of brisk lymphocytes, and susceptibility to immune checkpoint inhibitors (Haanen 2013; Huang and Zappasodi 2022).

The structural similarity of the RIPK4 kinase domain to both BRAF wild-type and V600 mutant proteins suggests its potential as a target for BRAF inhibitors (BRAFi). In the presence of vemurafenib or dabrafenib, the level of RIPK4 decreases regardless of the presence of BRAF mutations and independently of BRAF/ERK/MEK signaling (Madej et al. 2023). Reducing the level of a potential oncogene such as RIPK4, although it seems desirable, may result in other side effects. Therefore, studies investigating the functional role of RIPK4 in melanoma cells are important for a more comprehensive understanding of anti-melanoma treatment using BRAFi, as well as for elucidating the biology of this tumor.

In this study, we confirmed using RNA-seq analyses that RIPK4 is potentially involved in key biological processes such as cell adhesion, migration, proliferation, differentiation, and inflammatory processes in melanoma. We focused on RIPK4-dependent differentially expressed genes (DEG) associated with inflammation in WM266.4 melanoma cells which exhibit high levels of RIPK4 expression (Madej et al. 2021; Wronski et al. 2024). We suggest that RIPK4 may play a significant role in modulating the expression of BIRC3, IL-8, and IL-6 in response to TNF-α stimulation via the NFκB or/and p38/MAPK signaling pathways. For these studies, we used cell lines with silenced expression of RIPK4 achieved through both siRNA and stable downregulation using CRISPR/Cas9 techniques. In conjunction with a previous study, our data unveil the complex role of RIPK4 in regulating immune signaling networks in melanoma cells.

## Materials and methods

### Cell culture and treatment

The human melanoma cell line WM266.4 was kindly provided by the Department of Medical Biochemistry of the Jagiellonian University Medical College (Kraków, Poland) in 2006 and authenticated in 2021 using STR profiling with Identifiler Plus (ABI) and an ABI 3130xl Genetic Analyzer (Applied Biosystems, Waltham, MA, USA). Human melanoma cell line A375 was obtained from the American Type Culture Collection (CRL_1619, ATCC, Manassas, VA, USA) in 2020. Both cell lines were cultured in RPMI1640 containing 10% FBS (Gibco, Thermo Fisher Scientific, Waltham, MA, USA), penicillin 150 U/ml (Sigma-Aldrich, St. Louis, MO, USA), and streptomycin 100 µg/mL (Sigma-Aldrich, St. Louis, MO, USA) at 37 °C at 5% CO_2_ and 95% humidity. TNF-α (100 ng/mL, Sigma-Aldrich, St. Louis, MO, USA) was dissolved in 0.1% BSA/ PBS, aliquoted, and stored at − 20 °C. For ELISA analysis, the cell culture medium was replaced with serum-free medium and TNF-α (10 ng/mL) was added 24 h, while for qRT-PCR, TNF-α was added to the complete media for 3 h. The effect of p38 MAPK was studied using a p38 inhibitor SB203580 (10 μM, Sigma Aldrich, St. Louis, MO, USA) which was applied 1 h before TNF-α stimulation and present during the treatment.

### Immunostaining of melanoma section

Formalin-fixed paraffin-embedded 4 µm melanoma sections were used for both IHC (for RIPK4) and IF (for p65 NFκB subunit), as previously described (Janjetovic et al. 2011; Madej et al. 2021). The characteristic of melanoma patients is presented in Supplementary materials Table S1. The study was approved by the Committee of Ethics of Scientific Research of Collegium Medicum of Nicolaus Copernicus University, Poland (no. KB 448/2009). Briefly, after deparaffinization, rehydration, antigen retrieval in citrate buffer, or Tris/EDTA, sections were incubated overnight at 4 °C with primary antibody (Supplementary materials Table S2), followed by incubation with secondary antibody. IHC sections were assessed under the BX41 (Olympus Optical Co., Tokyo, Japan) microscope, ColorView III camera (Soft Imaging System, Hanover, Germany), and analySIS 3.2 software (Soft Imaging System). The sections were evaluated semiquantitatively, as previously described (Madej et al. 2021). Since we observed heterogeneous agranular and granular staining, the assessment was performed separately for each staining pattern. Sections for IF were incubated with AlexaFluor 488-conjugated secondary anti-rabbit antibody and mounted in Vectashield mounting medium with propidium iodide (PI) (Vector Laboratories Inc., Burlingame, CA, USA). Images (five for each sample) were recorded using BX-50 epifluorescence microscope (Olympus, Tokyo, Japan), FITC and TRITC filters, and Nikon DS digital camera. Images were merged using NIH ImageJ software. Staining intensity was scored as negative (0), weak (1), and strong (2). The number of p65 NFκB subunit-positive melanoma nuclei was counted, and the percentage of positive cells was calculated.

### Transfection with small interference RNA (siRNA)

Melanoma cells were seeded in 35-mm cell culture plates at a density of 3 × 10^5^ for 24 h before transfection. Transfection was carried out as previously described (Madej et al. 2021) using RIPK4-specific Silencer Select siRNAs (ID: s28865 Thermo Fisher Scientific, Waltham, MA, USA) or Silencer Select Negative Control No. 2 (cat. no. 4390846, Thermo Fisher Scientific, Waltham, MA, USA), as a control. The level of RIPK4 in cells transfected with siRNA was verified by Western blot or qRT-PCR analysis.

### CRISPR-Cas9 cell transduction

WM266.4 and A375 cells were co-transduced with lentiviral vectors encoding the Cas9 endonuclease (Invitrogen™, Thermo Fisher Scientific, USA) and single-guide RNA (sgRNA) either complementary to a fragment of the RIPK4 gene (ID: CRISPR1029813_LV; Invitrogen™, Thermo Fisher Scientific, USA) or lacking homology with a region of the human genome (as a negative control; cat. no. A32063; Invitrogen™, Thermo Fisher Scientific, USA) as described previously (Wronski et al. 2024). In addition, the control vector construct encoded a GFP protein. Stable silencing (knockout) of RIPK4 in cells was evaluated by Western blot.

### Cytokine analysis in human serum

The serum was collected from melanoma patients qualified for radical radiotherapy to the area of regional lymph nodes and from control group consisted of patients with non-melanoma tumors (as non-neoplastic lesions, epidermal nevi, basal cell cancers, seborrheic verrucas, lentigo simplex, hemangioma, eratopapilloma) qualified for surgical removal of the lesion. The characteristic of patients included in this study is presented in Supplementary materials Table S3. The whole blood samples were collected into tubes containing a clot activator (SST, Vacutainer SST II Tube 8.5 mL, Becton Dickinson, Sunnyvale, CA, USA) and incubated undisturbed at room temperature for 20–30 min, followed by centrifuging at 2000 × *g* for 10 min at 4 °C. The serum (supernatant) was aliquoted (500 µl) into 1.5 mL cryovials, frozen, and stored at -80 °C until analysis. Human Cytokine/Chemokine Magnetic Bead Panel (cat. no. HCYTOMAG-60 K, Merck Millipore, Burlington, MA, USA) was used for the evaluation of TNF-α, IL-13, IL-1b, IL-2, IL-4, and IL-6 concentrations in serum with Luminex xMAP^®^ technology. The procedure was performed as previously described (Deptuła et al. 2020; Wardowska et al. 2020), according to manufacturer’s instructions. Briefly, samples ware thawed on ice and incubated overnight with a mixture of antibody-immobilized color-coded beads. After incubation, the plate was washed, and biotinylated detection antibodies specific for analytes of interest were introduced for 1 h. Next, streptavidin–phycoerythrin (PE) conjugate, which binds to detection antibodies, was added for 30 min. Then, the plate was washed and read by a Luminex MAGPIX^®^ Analyzer (Merck Millipore, Burlington, MA, USA). The obtained data were analyzed with PONENT 4.2 software and presented as pg/ml. Since for more than 75% of cases the level of IL-13, IL-1b, IL-2, IL-4, and IL-6 cytokines was under the detection point, only TNF-α was statistically analyzed.

### RNA isolation

Total RNA was isolated for sequencing and real-time PCR using Total RNA Mini Plus (A&A Biotechnology, Gdańsk, Poland) according to the manufacturer’s recommendations. Total RNA concentration and purity were determined by measuring absorbance at 260/280 nm and 260/230 nm using a CLARIOstar Plus spectrophotometer (BMG LABTECH, Ortenberg, Germany).

### Total RNA sequencing (RNA-seq)/next-generation sequencing analysis

Total RNA sequencing (RNA-seq) was performed to analyze global gene expression changes after transfection of cells with siRIPK4 or negative si-control (neg.si). The library preparation, RNA-seq, and RNA-seq data preparation were performed by Genomed (http://www.genomed.pl/, Warszawa, Poland) according to the manufacturer’s instructions for the BGISEQ500 platform (BGI Genomics, Shenzhen, China) and were performed in triplicate. The service was performed using BGI’s reagents and included polyA fraction enrichment, single strand circular (ssCir DNA) library construction and paired-end sequencing (PE100, 30M) using a DNA nanoball sequencing (DNBSEQ™) technology. The first step in the data analysis was to remove the adapters using the Cutadapt package (Martin 2011). Filtering was performed using the quality parameter *q* 25 and the minimum length of the reading *m* 15. Quality reports were generated using FASTQC software (Andrews 2010). The reads were mapped with TopHat (Trapnell et al. 2012) to the reference genome of Homo sapiens in the version GRCh38.p13 (https://www.gencodegenes.org/human/). TopHat was launched with the option to prepare the fr-unstranded library and in the no-new-juncs mode. Then, the number of pairs of readings assigned to individual genes was counted using HTseq (Anders et al. 2015) without differentiation regarding the strand of the transcript (–stranded = no). Differential expression analysis of three groups (three biological replicates per condition) was performed using the DESeq2 R package (1.36.0) (Love et al. 2014). The resulting P values were adjusted using the Wald test approach to control the false discovery rate (FDR). Functional enrichment analysis including gene ontology (GO) analysis was conducted to identify DEGs which were significantly involved in each GO term. GO enrichment analysis was performed using BiNGO (v3.0.4, in Cytoscape 3.8.0) (Maere et al. 2005); GO term that exhibited a significant enrichment with FDR < 0.05 was considered. Furthermore, the DEBrowser platform (Kucukural et al. 2019) was used to obtain a graphical representation of the DEGs on the heatmaps.

### Construction of the protein–protein interaction (PPI) network

The database of Interacting Genes/Proteins Search Tool for the Retrieval of Interacting Genes/Proteins (STRING; www.string-db.org) database (Szklarczyk et al. 2021) and the GeneMANIA website (http://genemania.org) (Warde-Farley et al. 2010) were used to predict functionally similar genes of hub genes and construct the PPI network among them. It can also indicate the relationships among functionally similar genes and hub genes, including physical interaction, signaling pathways, and colocalization.

### qRT-PCR

Quantitative real-time PCR was performed to validate the reliability of the RNA-seq gene expression data. One microgram of RNA was reverse transcribed into cDNA using oligo(dT)18 and the TranScriba kit (A&A Biotechnology, Gdańsk, Poland). qRT-PCR reactions were performed using the Sensitive RT HS-PCR Mix (A&A Biotechnology, Gdansk, Poland) and the qTOWER3 real-time PCR thermal cycler (Analytik Jena, Jena, Germany). All TaqMan primers were purchased from Thermo Fisher Scientific/Invitrogen, Waltham, MA, USA and included in Supplementary Materials Table S4. The relative levels of the transcripts were quantified by the 2^−ΔΔCt^ method. GAPDH was used for standardization.

### Immunoblotting

Cells were lysed in RIPA buffer supplemented with freshly added protease and phosphatase inhibitors (Sigma-Aldrich, St. Louis, MO, USA). Twenty micrograms of protein evaluated using the BCA method (Sigma-Aldrich, St. Louis, MO, USA) was loaded onto a 12% SDS polyacrylamide gel (Bio-Rad, Hercules, CA, USA) followed by electrophoresis at a constant voltage of 100 V for 1.5 h. Proteins were transferred to a PVDF membrane (0.2 μm pore size, Merck Millipore, Burlington, MA, USA). The membranes were incubated for 40 min in blocking solution: 4% bovine serum (BSA; Sigma-Aldrich, St. Louis, MO, USA) in TBS-T buffer (25 mM Tris, 0.2 M glycine, 20% methanol, 1% Tween) and incubated overnight at 4 °C with primary and then with secondary antibodies (Supplementary Materials Table S2). The detection was performed using a Clarity Western ECL substrate (Bio-Rad, Hercules, CA, USA) with the ChemiDoc detector (Bio-Rad, Hercules, CA, USA). Protein band intensities were quantified using ImageLab 5.2.1 software.

### ELISA

The human IL-8 ELISA set (BD OptEIA™, BD Biosciences, San Jose, CA, USA) and IL-6 (DY206 from R&D systems, Minneapolis, MN, USA) were used to determine the secretion of IL-8 or IL-6 in the culture medium by melanoma cells, which was performed according to the manufacturer’s instructions. The optical density of each well was determined using a LEDETECT96 microplate reader (Labexim Products, Lengau, Austria). IL-8 and IL-6 concentrations in the medium samples were obtained by fitting a four-parameter logistic (4-PL) curve.

### Statistics

Data were presented as mean ± SD unless otherwise indicated in the figure legends. The statistical tests used are indicated in figure legends. Statistical analyses were performed using GraphPad Prism (GraphPad Software Inc., San Diego, CA), expect for ELISA results which were analyzed using STATISTICA v.13 (StatSoft, Inc.). Differences between the two groups were evaluated using either the Student’s *t* test or the Mann–Whitney test. For multiple comparisons, a one-way analysis of variance (ANOVA) was employed for statistical analysis. A *p* value < 0.05 was considered statistically significant.

## Results

### Global analysis of changes in gene expression profile under downregulation of RIPK4

**Fig. 1:**
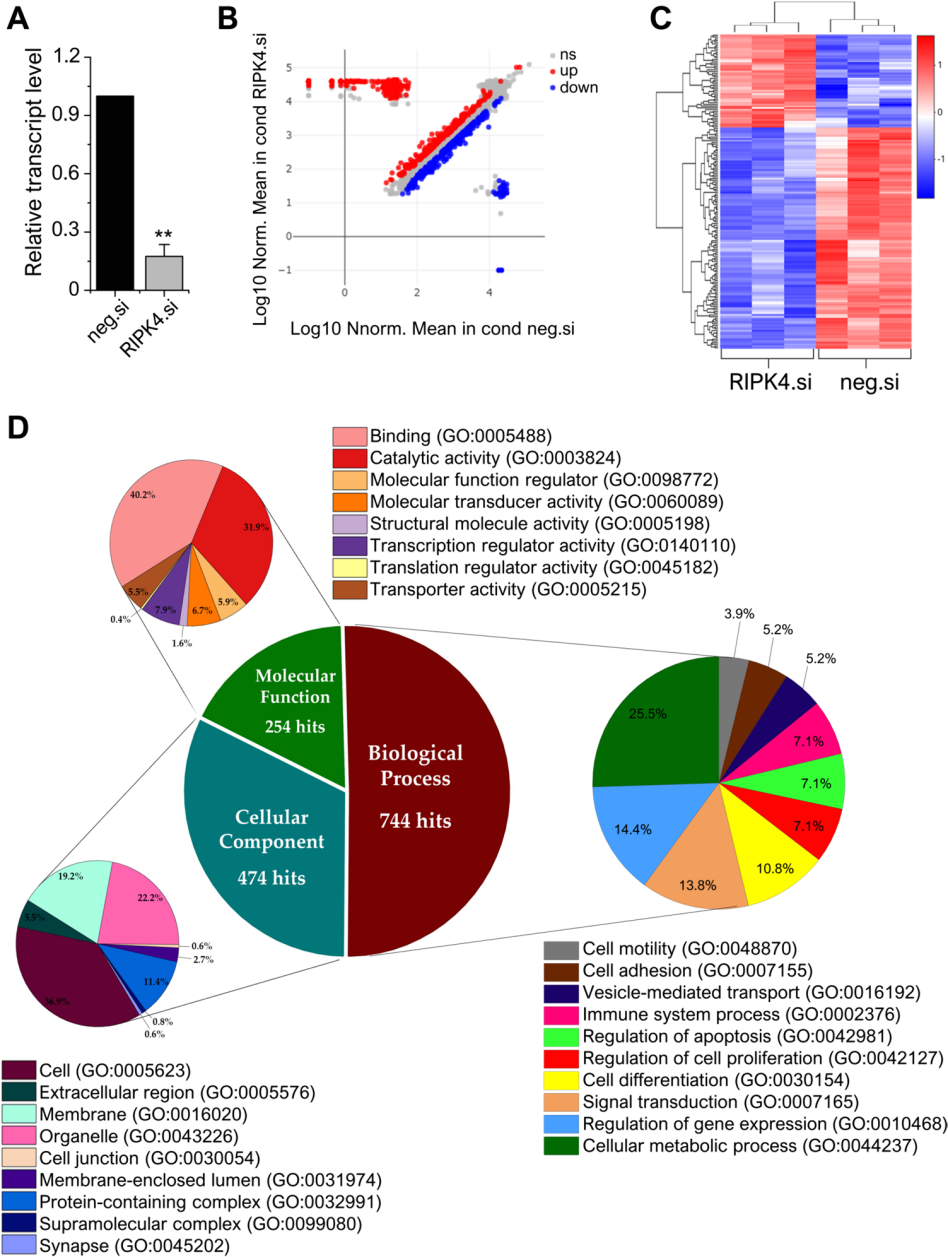
RNA-seq analysis revealed significant changes in gene expression under downregulation of RIPK4 in WM266.4 cells. RIPK4 expression in cells transfected with neg.si and RIPK4.si was analyzed by qRT-PCR 48 h after transfection. The transcription level of RIPK4 was normalized to GAPDH (**A**). A scatter plot showing the correlation of transcript expression profile between RIPK4 silenced cells (RIPK4.si) and control cells (neg.si) using the DEBrowser platform (**B**). A heatmap showing the expression levels of significantly (FDR-adjusted *p* < 0.5) differentially expressed genes after transfection. The rows and columns were clustered based on similarity in predicted expression patterns. The red-blue gradient indicates the expression of the transcript in three independent experiments. Red = highest expression; blue = lowest expression (**C**). Classification of DEGs between RIPK4 silenced cells (RIPK4.si) and control cells (neg.si) based on their functions (molecular functions, cellular components, and biological processes) using BiNGO tool as a Cytoscape plugin. The percentages were calculated as the number of gene hits to the total number of hits in each category (**D**)

RNA sequencing technology (RNA-Seq) was used to characterize global transcriptome changes after silencing of RIPK4 in WM266.4 melanoma cells, in which the transfection efficiency was approximately 80% (Fig. 1A). The tested WM266.4 cell line exhibited high expression of this kinase, as indicated by our previous data (Madej et al. 2021, 2023). After 48 h of cell transfection, with siRNA specific for RIPK4 or negative siRNA as a control, isolated RNA was sequenced using the BGISEQ500 platform. Sequencing data can be accessed through NCBI GEO with an accession number (GSE 263112 available after July 1, 2024).

Analysis of RNA-seq data using the DEBrowser v1.22.4 platform demonstrated significant changes in the transcript expression profile between RIPK4.si and control cells (neg.si) (Fig. 1B). Silencing RIPK4 led to 1810 differentially expressed transcripts with a change of at least 50%, of which 613 were statistically significant (*p* < 0.05), and 407, including correction for multiple tests (pFDR < 0.05). The 407 identified significantly differentially expressed genes (DEG) are shown in the heatmap in Fig. 1C.

**Fig. 2:**
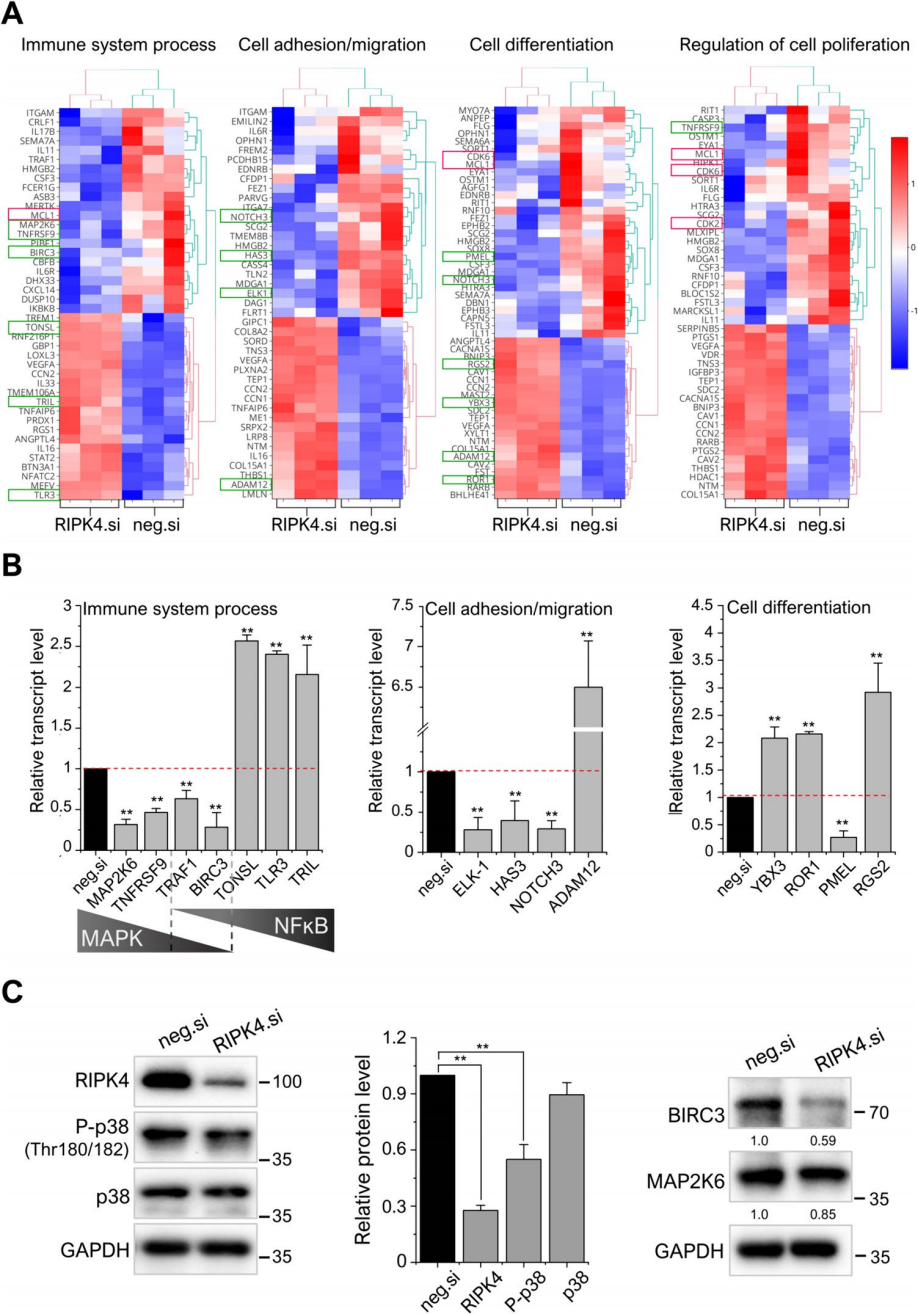
Differentially expressed transcripts upon RIPK4 silencing in WM266.4 cells. Heatmap of DEGs in selected biological processes as immune system, cell adhesion/migration, cell differentiation, and cell proliferation associated with RIPK4. Red-blue gradient indicates the expression of transcript in three independent experiments: red = highest expression; blue = lowest expression. Selected processes were assigned to specific transcripts using Cytoscape platform (BiNGO apps). Genes selected for quantitative analysis with RT-PCR are marked with green squares (**A**). Validation of selected transcripts by qRT-PCR. RIPK4.si transfected cells were compared to neg.si samples. Relative mRNA levels of indicated transcripts were normalized to GAPDH. n = 3. **p < 0.001 (**B**). The expression levels of the P-p38, p38, BIRC3, and MAP2K6 proteins were analyzed using Western blotting along with densitometry. [*n* = 1–3] (**C**)

Gene ontology (GO) analysis (Fig. 1D) using BiNGO plugin in Cytoscape software revealed that DEGs belong to the “cell” and the “organelle” categories which were highly represented in the category of cellular components. In the Molecular Function category, we observed  an enrichment of genes in the “bidding” and “molecular activity” clusters. Interestingly, when analyzing GO terms describing biological processes, DEGs were enriched in transcripts involved in “cellular metabolism” and “regulation of gene expression,” as well as terms related to cancer progression such as “motility/adhesion,” “immune system process,” “cell differentiation,” “regulation of apoptosis,” and “proliferation” clusters. We previously observed that RIPK4 downregulation inhibits proliferation, migration, and tumor growth in WM266.4 and A375 cells (Madej et al. 2021, 2023; Wronski et al. 2024). In the current study, we identified DEGs associated with these key biological processes: adhesion/migration (41 genes), differentiation (52 genes), cell proliferation (45 genes), and inflammation-related genes (42 genes). These findings are visualized as heatmaps in Fig. 2A.

To validate the accuracy of the transcriptome sequence data, we selected 16 genes (highlighted by green squares) involved in multiple processes crucial for tumorigenesis for quantitative RT-PCR analysis. It should be noted that some transcripts can be assigned to more than one process. As shown in Fig. 2B, the transcriptome data were consistent with the results of quantitative RT-PCR. The silencing of RIPK4 led to decreased levels of MAP2K6, TNFRSF9, TRAF1, and BIRC3 transcripts, along with increased levels of TONSL, TLR3, and TRIL transcripts. These transcripts encode proteins involved in NFκB and MAPK associated with immune response processes. Notably, MAP2K6, MAP2K3, and IL-6R, which belong to the MAPK kinase pathway, are the main activators of p38 and JNK (Darling and Cook 2014). Furthermore, our results showed a decrease in the expression of ELK-1, HAS3, NOTCH3, and PMEL genes along with an increase in ADAM12, YBX3, ROR1, and RGS2 genes upon RIPK4 downregulation.

The silencing of RIPK4 resulted in significant changes in the transcriptome, accompanied by 40% decrease in the phosphorylation level of the p38 protein compared to the control. In addition, we observed a substantial 40% decrease in the protein level of BIRC3, while the MAP2K6 protein level remained slightly changed (Fig. 2C). Although previous studies have indicated the involvement of RIPK4 in adhesion, migration, differentiation, and genes associated with inflammatory processes (Adams et al. 2007; Holland et al. 2002; Kwa et al. 2014, 2016), these have not been demonstrated specifically in melanoma.

### RIPK4 interaction with DEGs associated with inflammation-related genes

**Fig. 3:**
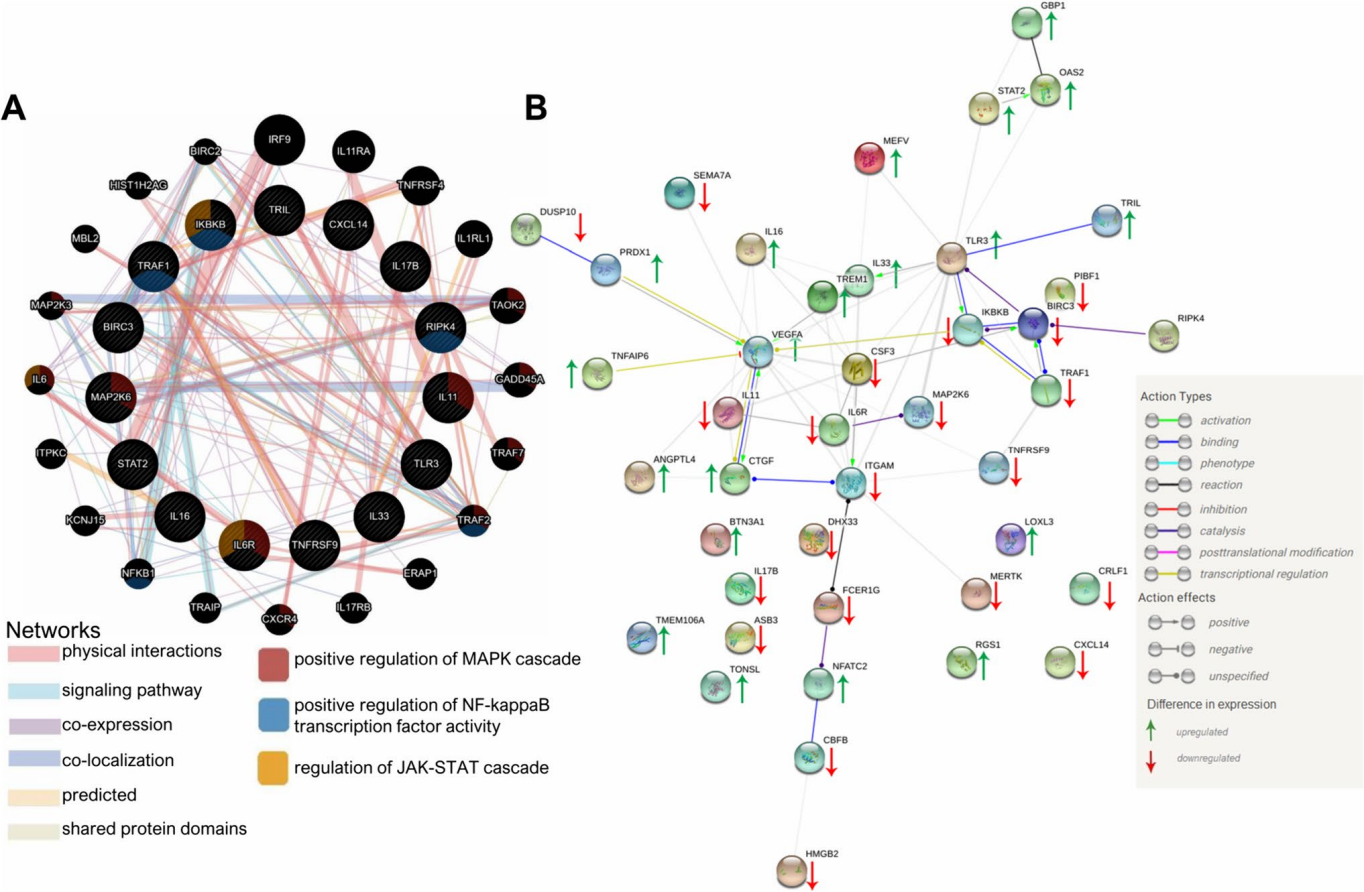
A network of interactions between genes involved in the regulation of inflammation, in which transcript levels were altered by downregulation of RIPK4 expression in WM266.4 cells. The analysis was performed in the GeneMANIA (**A**) and STRING (**B**) databases. The network shows the following types marked according to the legend, interactions: activation, binding, catalysis, transcriptional regulation

As melanoma is one of the immunogenic neoplasms, we focused on genes related to inflammation. Based on the STRING and GeneMANIA databases, we constructed a protein–protein interaction network of selected DEGs linked to inflammation (Fig. 3). Notably, 10 out of the 41 DEGs did not exhibit connections within any type of network (STRING interaction score = 0.4).

Thirty DEGs were connected to networks through complex relationships. The functional partner of RIPK4 predicted by the STRING tool is BIRC3. BIRC3 in turn forms a further network of interactions with TRAF1, IKBKB, and TLR3 and indirectly with TRIL and IL-33. Furthermore, BIRC3 is connected to TRAF1, which physically interacts with TNFRSF9. TRAF1 and TNFRSF9 are receptors for TNF-α (Rothe et al. 1994; Snell et al. 2011). In addition, catalysis between IL-6R and MAP2K6 is observed separately (Fig. 3B). The interaction network obtained using STRING indicates that MAP2K6 kinase is regulated by IL-6R activation, while the GeneMANIA database showed a close functional relationship between MAP2K6 and MAP2K3. Using database resources, additional information was also obtained on the gene membership of selected signaling pathways important for inflammation regulation. RIPK4 is predicted to be part of the NFκB transcription factor, along with TRAF1 and IKBKB (Fig. 3B).

Bioinformatics analysis revealed two additional pathways: the MAP kinase cascade and the JAK-STAT signaling pathway (Fig. 3A). However, genes associated with the JAK-STAT cascade did not exhibit significant alterations in expression in the NGS analysis (*p* > 0.05, Supplementary Materials Table S5). A better understanding of the role of RIPK4 in regulating the JAK-STAT cascade requires further study. The outer circle of the signaling network includes genes not directly analyzed but identified by the database as closely related to the studied genes (Fig. 3A). This provides important information for planning further experiments and better understanding the impact of RIPK4 on inflammation regulation.

**Fig. 4:**
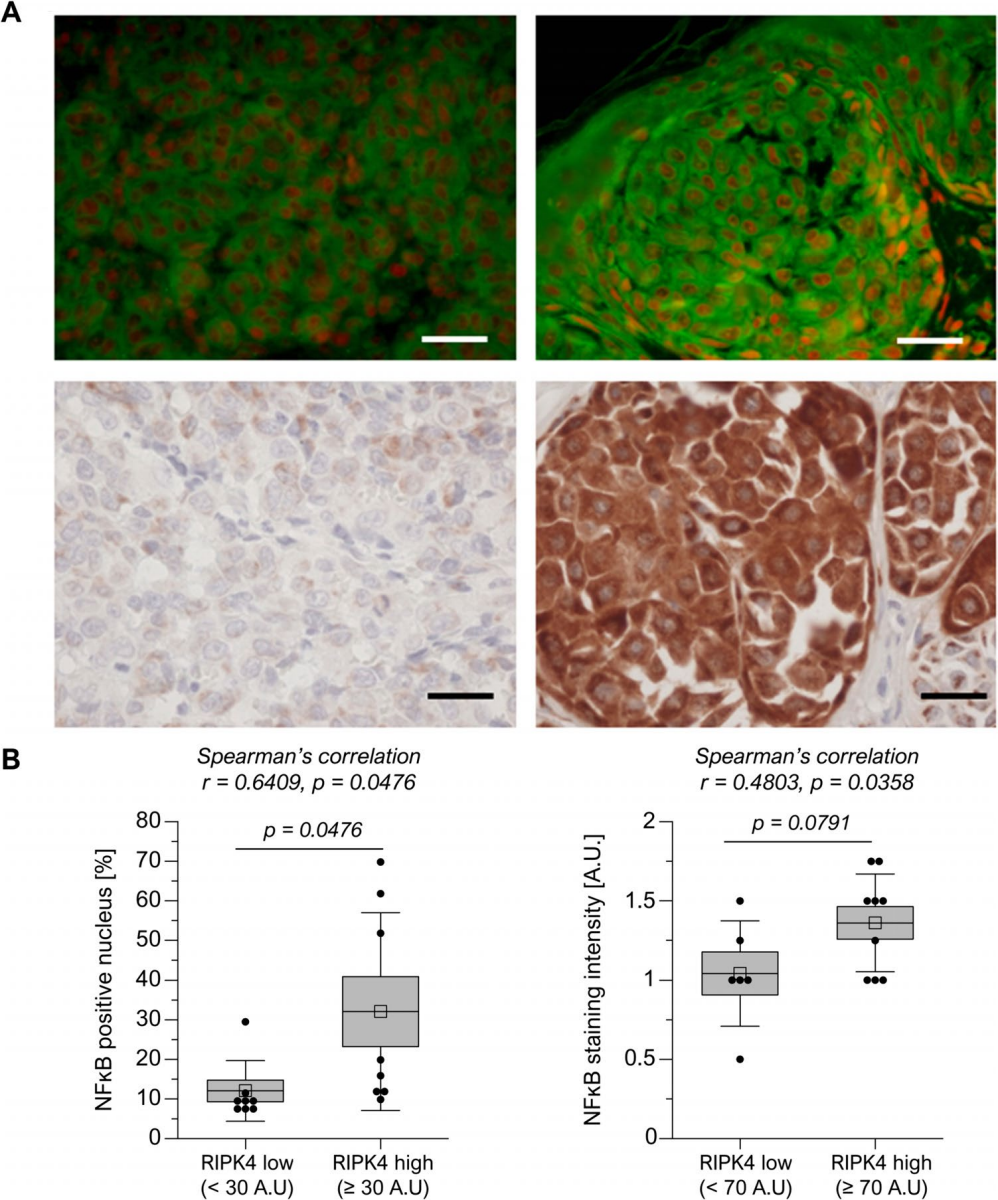
Relationship between NF-κB and RIPK4 expression in cutaneous melanomas (*n* = 17). p65 subunit of nuclear factor-κB immunofluorescence (upper row) in RIPK4 (lower row) showing low (left column) and high (right column) expressions in human cutaneous melanomas. p65 (AlexaFluor 488-green) and nuclei (PI-res) signals were captured using epifluorescence microscope with 475 nm and 542 nm excitation wavelengths, respectively, and merged. Scale bars = 50 μm (**A**). Percentage of p65 subunit of NFκB-positive melanoma cells nuclei (left) and NFκB staining intensity (right) in melanoma cells in melanoma cases showing RIPK4 low and RIPK4 high agranular and granular staining, respectively. Low and high expressions of RIPK4 were distinguished with the median value. Spearman’s correlation between NFκB and RIPK4 is indicated in the graph (**B**)

Our previous studies have demonstrated that downregulation of RIPK4 by siRNA decreases the level of P-p65 in WM266.4 and A375 cells (Madej et al. 2021). To explore the relationship between NFκB and RIPK4 expression, we performed immunohistochemical analysis of RIPK4 levels along with immunofluorescence for the p65 subunit of NFκB in clinical specimens of cutaneous melanomas (Fig. 4A). The positive correlation observed between RIPK4 expression levels and the number of p65-positive cells, as well as the intensity of p65 fluorescence in melanoma biopsies (Fig. 4B), further confirms the link between RIPK4 expression and NF-κB activation.

### RIPK4 downregulation inhibits TNF-α-stimulated IL-8 and IL-6 production in melanoma cells

**Fig. 5:**
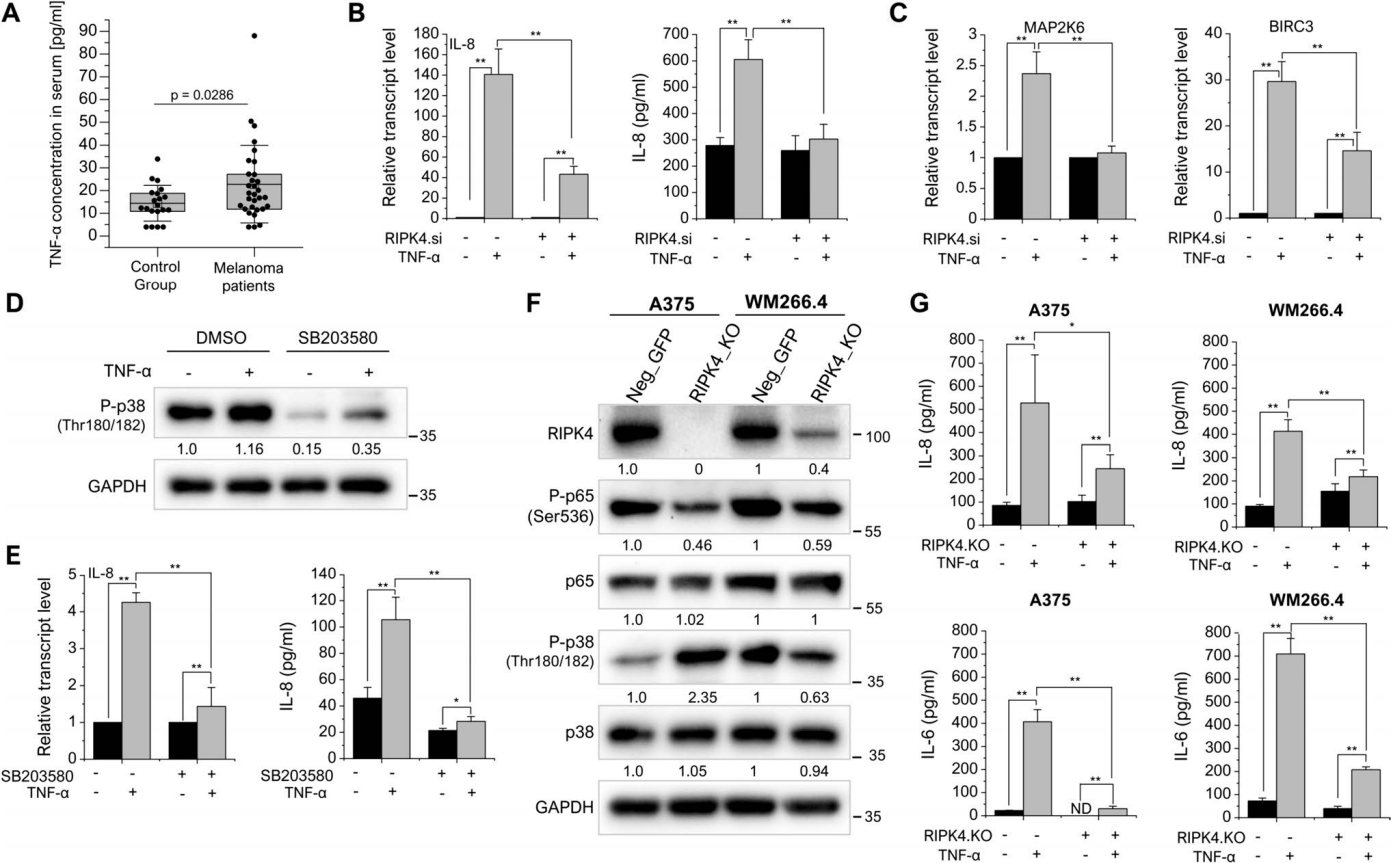
Effect of downregulation of RIPK4 on selected protein expression after TNF-α stimulation. TNF-α concentration in the serum in melanoma patients (*n* = 32) and control group (*n* = 20). The p value represents statistical significance in Mann–Whitney test (**A**). Effect of TNF-α (10 ng/ml) in RIPK4.si and neg.si transfected cells on mRNA and secretion level of IL-8 (**B**) and mRNA level of MAP2K6 and BIRC3 transcripts normalized to GAPDH. *n* = 3, **p < 0.01. (**C**). Effect of TNF-α (10 ng/ml) on pre-treated WM266.4 cells with SB203580 (10 µM) on RIPK4 and P-p38 expression assessed by Western blot (**D**) and mRNA and secretion level of IL-8 (**E**). Selected protein levels in A375^RIPK4.KO^ and WM266.4^RIPK4.KO^ cells along with densitometry. GAPDH served as loading control. (**F**). Effect of TNF-α on IL-8 and IL-6 secretion in A375^RIPK4.KO^ and WM266.4^RIPK4.KO^ cells and their controls. *n* = 3, ND indicates not determined (**G**)

TNF-α is one of the pleiotropic cytokines, playing a central role in the tumor-associated cytokine network. In addition, TNF-α stimulates the secretion of IL-8 by NF-κB activation (Osawa et al. 2002), and its effects on melanoma growth and metastasis have been extensively reviewed in previous literature (Anghel et al. 2015; Bar-Eli 1999; Singh and Varney 2000). Therefore, we evaluated TNF-α levels in the serum of patients with advanced melanoma. The mean levels of TNF-α in patients’ serum were significantly higher (33.8 pg/ml; *n* = 32), compared to healthy volunteers (22.8 pg/ml; *n* = 20) (Fig. 5A).

Furthermore, to investigate the impact of RIPK4 on IL-8 expression, we downregulated RIPK4 in WM266.4 cells and subsequently treated them with TNF-α. Our findings revealed a significant suppression of IL-8 at both the mRNA level (70% reduction, *p* < 0.01) and in secretion (87% reduction, p < 0.01) (Fig. 5B). These observations were accompanied by a decrease in mRNA expression of BIRC3 and MAP2K6 (Fig. 5C). Since we demonstrated that RIPK4 inhibits p38 phosphorylation, we used an inhibitor of p38 activation (SB203580) and assessed its effect on TNF-α-stimulated IL-8 production. We found inhibition of p38 phosphorylation and IL-8 production (Fig. 5D and E), confirming that RIPK4 may play a role in IL-8 secretion via p38 pathway. Interestingly, TNF-α increased the expression of RIPK4 independently of the presence of p38 phosphorylation inhibitors.

To explore the long-term effects of RIPK4 silencing and overcome the limitations of transient siRNA-mediated knockdown, we performed stable silencing of RIPK4 using the CRISPR-Cas9 system. We generated two melanoma cell lines, WM266.4 and A375, with confirmed *RIPK4* knockout (RIPK4.KO) along with their respective control cells (Neg.GFP) (Fig. 5F). Assessing the impact of stable RIPK4 silencing on cytokine production, we stimulated the RIPK4.KO and Neg.GFP cells with TNF-α and measured IL-8 production using ELISA. In addition, the levels of IL-6, which is one of the most induced NFκB-dependent cytokines (Brasier 2010), were examined. Figure 5G shows that both A375^RIPK4.KO^ stimulated with TNF-α and WM266.4^RIPK4.KO^ produced less IL-8 and IL-6 than those of their Neg.GFP controls.

## Discussion

RIPK4 is involved in many processes related to skin development and epidermal homeostasis, as it is required for keratinocyte differentiation. Recent studies have shown that RIPK4 plays an important role in various cancers, exhibiting both oncogenic (Gong et al. 2018; Liu et al. 2015, 2018, 2021; Qi et al. 2018; Yi et al. 2020) and tumor suppressive (Kopparam et al. 2017; Li et al. 2021; Poligone et al. 2015; Wang et al. 2014) functions, depending on the cellular context. In our prior study, we demonstrated heterogeneous expression of RIPK4 in melanoma samples, where it acts as an oncogene, in contrast to cutaneous squamous cell carcinoma, where it functions as a tumor suppressor (Madej et al. 2021). To investigate the molecular function of the RIPK4 protein in melanoma cells, we performed a transcriptome analysis of WM266.4 cells with silencing of this kinase.

The transcriptome analysis revealed the regulatory role of RIPK4 in the invasive potential of melanoma cells by participating in the regulation of the expression of numerous genes crucial for metastasis. Gene ontology analysis of DEGs based on biological processes indicates that both downregulated and upregulated DEGs were associated with adhesion/migration, differentiation, cell proliferation, and inflammation. These findings align with the essential role of RIPK4 in normal keratinocytes (Adams et al. 2007; Holland et al. 2002; Kwa et al. 2014, 2016).

Global transcriptome analysis of WM266.4 melanoma cells with silencing of RIPK4 confirmed the significant role of RIPK4 kinase in melanoma cell transformation and development. This was demonstrated by changes in the expression levels of genes associated with cell adhesion, the formation of adhesion foci, and the dynamics of the architecture of the actin cytoskeleton and extracellular matrix. Notably, our previous observations indicated that RIPK4 silencing led to alteration in focal contact architecture, resulting in reduced cell movement of melanoma cells (Madej et al. 2021). Furthermore, in the present analysis, we found that RIPK4 may be involved in regulating the expression levels of hyaluronan synthase 3 (HAS3), Notch receptor 3 (NOTCH3), and ADAM12, which are important for cell migration (Arasu et al. 2020; Howard et al. 2013; Pekkonen et al. 2018; Skandalis et al. 2020; Thodeti et al. 2005). Overexpression of the ETS-domain containing protein (ELK-1) promoted the adhesion, migration, and invasion of colorectal cancer (Ma et al. 2021). This transcription factor has been reported to be directly phosphorylated by p38 (Ferreiro et al. 2010). It appears that RIPK4 kinase may regulate the adhesive properties of melanoma cells by involving in the p38/EKL-1 axis.

In addition, the analysis confirms our previous findings that silencing RIPK4 in WM266.4 cells significantly reduces their proliferative potential. This effect is attributed to the inhibition of the cyclin-dependent kinase pathway CDK2/CDK6, suggesting impaired proliferation rather than increased cell death as the underlying mechanism (Madej et al. 2021, 2023). Interestingly, proteins responsible for regulating cell adhesion, such as NOTCH3 (Pekkonen et al. 2018) and ADAM12 (Roy et al. 2017), are known to also play a role in controlling cell proliferation. The analysis also revealed a significant increase in the expression of RGS2, a member of GTPase-activating protein family, upon RIPK4 silencing. This upregulation of RGS2 inhibits MAPK and AKT signaling, thereby preventing melanoma cell growth, as demonstrated by Lin et al. (Lin et al. 2021). Consistent with our previous findings, we noted a significant reduction in the activation of the AKT/FAK signaling axis in WM266.4 cells with a decreased level of RIPK4 (Madej et al. 2023), which may be a result of induced RGS2 expression.

Increased production of proinflammatory cytokines by melanoma cells and persistent chronic inflammation in their microenvironment have been shown to promote tumor progression and metastasis (Filimon et al. 2021; Simiczyjew et al. 2020). In keratinocytes, RIPK4 and IRF6 function as a cell-intrinsic signaling axis that not only regulates keratinocyte differentiation but also mediates the expression of proinflammatory cytokines such as CCL5 and CXCL11 (Kwa et al. 2016). In addition, studies suggest that RIPK4, acting through IRF6, may regulate ELOVL4 to control lipid and fatty acid metabolism (Oberbeck et al. 2019; Yan et al. 2023). Botti et al. reported a tumor-suppressive function of IRF6 in squamous cell carcinomas (Botti et al. 2011); however, studies on melanoma are limited. Our results reveal that IRF6 expression in the WM266.4 cell line is very low. The expression of ELOVL4 is relatively higher, but we did not observe a statistically significant difference between cells with silencing RIPK4 and control cells (*p* = 0.94). This suggests a relatively minor impact of IRF6 and ELOVL4 in melanoma, further emphasizing the context-dependent role of RIPK4 in both non-melanoma and melanoma skin cancers.

Our study reveals a possible association of inflammation-related DEGs and the expression of RIPK4 in melanoma, which has not been previously described. Using the STRING and GeneMANIA databases, which generate predictions of gene interaction networks, revealed the potential involvement of RIPK4 kinase in regulating inflammation through the NF-κB and MAPK pathways. This observation is in concordance with our previous report, showing that RIPK4 kinase regulates NF-κB signaling pathway in melanoma cells, thereby enhancing their invasive potential (Madej et al. 2021). Herein, we confirm the correlation of RIPK4 expression with the number of p65-positive cells as well as the intensity of p65 fluorescence in clinical samples.

The STRING tool predicts BIRC3 as the functional partner of RIPK4. According to Bertrand et al., RIPK4 plays a role as a mediator in the regulation of the NF-κB pathway by interacting with BIRC3, a protein known as an apoptosis inhibitor that forms a complex with the TRAF protein (Bertrand et al. 2011). It is well established that the expression of BIRC3 could be stimulated by TNF-α (Diessenbacher et al. 2008). Our findings notably indicate an increase in TNF-α concentration in serum in advanced melanoma patients, confirming studies by Wang et al., where TNF-α has been implicated in melanoma progression, positively correlated with Breslow thickness and serum levels of TNF-α (Wang et al. 2021).

TNF-α is a multifunctional cytokine that plays a role in inflammation, immunity, antiviral responses, and various diseases. Its pleiotropic action modulates the tumor microenvironment through paracrine mechanisms in the context of tumor tissue. Our data suggest that downregulation of RIPK4 decreases the expression of BIRC3 and MAP2K6 in cells stimulated by TNF-α. Osawa showed that the induction of IL-8 expression by TNF-α could be inhibited when two survival signals. NF-κB and PI3K/Akt, were suppressed by the presence of mutant form of inhibitor of NF-κB (IκB); by dominant negative (kinase-dead) Akt; or by treating cells with LY 294002, an inhibitor of PI3K (Osawa et al. 2002). Interestingly, we have already revealed that RIPK4 downregulation impairs NF-κB and PI3K/Akt signaling (Madej et al. 2021, 2023).

In this study, we have found that downregulation of RIPK4 using two different techniques, siRNA and CRISPR/Cas9, inhibits IL-8 and IL-6 production in two melanoma cell lines, WM266.4 and A375. Singh et al. showed a positive correlation between the level of IL-8 produced by melanoma cells and their metastatic potential (Singh et al. 2010). These observations under in vivo conditions were confirmed by Huang et al. (Huang et al. 2002), using ABX-IL-8 antibodies that inhibit the interaction of IL-8 with its receptor as a therapy against melanoma. Their study also confirmed that IL-8 inhibition significantly reduces the expression of extracellular matrix metalloproteinase 2 (MMP-2), thereby reducing the angiogenic potential of melanoma cells and providing lung metastasis. The expression of cytokines and other inflammatory mediators is subject to regulation involving various molecules, including RIPK4 kinase (Kwa et al. 2016). The primary role of this protein is regulating the differentiation process of keratinocytes. However, it has also been shown to have a role in enhancing the expression of proinflammatory cytokines such as IL-8 and CCL5 through activation of the NF-κB and IRF6 pathways in these cells (Kwa et al. 2016).

We postulate that the attenuated signal transduction triggered by TNF-α is a consequence of the reduced expression of the TRAF1 and TNFRSF9 receptors. Tyciakova observed that TNF-α overexpression is accompanied by a significant upregulation of the proinflammatory cytokine IL-6 gene in A375 melanoma cells and proapoptotic ligand TRAIL gene in colorectal cancer cell HT29, both of which were mediated by TNF-α/TNFR1 signaling (Tyciakova et al. 2021). On the contrary, our study suggests that RIPK4 may regulate IL-8 expression by involving in the MAPK kinase cascade and NF-κB. Brancho et al. (Brancho et al. 2003) described TNF-dependent activation of the p38 pathway by MAP2K6 and MAP2K3. The p38 MAPK pathway, when activated, can phosphorylate a wide range of proteins and lead to the maintenance of an aggressive tumor phenotype and/or resistance to chemotherapy (Flem-Karlsen et al. 2019).

Summarizing, our recent data confirm our previous findings, indicating a correlation between NF-κB activation and RIPK4 expression in melanoma patient specimens. Furthermore, based on our new findings, it suggests that RIPK4 may play a pivotal role in regulating the expression of IL-8 and IL-6 in response to TNF-α stimulation of melanoma cells. This regulatory function of RIPK4 likely involves signal transduction through the BIRC3/p65 and p38 MAPK signaling pathway. These data reveal a complex role for RIPK4 in regulating the immune signaling network in melanoma cells and suggest that this kinase may serve as a potential complementary therapy for melanoma-targeted adjuvant therapy.

## Supplementary Information

Below is the link to the electronic supplementary material.

Supplementary file1 (DOCX 42 KB)

## Data Availability

Data will be made available on request.

## Abbreviations

BRAFi: BRAF inhibitors
CRISPR/Cas9: Clustered regularly interspaced short palindromic repeats/CRISPR-associated protein 9
DEG: Differentially expressed genes
IL: Interleukin
RIPK4: Receptor-interacting serine/threonine kinase 4
TNF-α: Tumor necrosis factor-alfa
RNA-seq: RNA-sequencing
