# Supplementary material for: The involvement of RIPK4 in TNF-α-stimulated IL-6 and IL-8 production by melanoma cells
Source: J Cancer Res Clin Oncol. 2024 Apr 24;150(4):209. doi: 10.1007/s00432-024-05732-3 (PMC11043103; doi:10.1007/s00432-024-05732-3)
Supplement: Supplementary file 1 — Supplementary file1 (DOCX 42 KB) [file 432_2024_5732_MOESM1_ESM.docx]

*Journal of Cancer Research and Clinical Oncology*

**Supplementary materials**

RIPK4 downregulation in melanoma cells impairs TNF-α- stimulated IL-8 production via p38.

Ewelina Madej^1^, **Anna Lisek^1^,** Anna A. Brożyna^2^, **Agnieszka Cierniak^3^**, Norbert Wronski^1^, **Milena Deptuła^4^, Anna Wardowska^5^,** Agnieszka Wolnicka-Glubisz^1^

^1^Department of Biotechnology, Jagiellonian University, Krakow, Poland

^2^Department of Human Biology, Nicolaus Copernicus University, Torun, Poland

^3^Department of Biochemistry, Faculty of Medicine and Health Sciences, Andrzej Frycz Modrzewski Krakow University, Kraków, Poland

^4^Laboratory of Tissue Engineering and Regenerative Medicine, Division of Embryology, Faculty of Medicine, Medical University of Gdansk, Poland,

^5^Department of Physiopathology, Faculty of Medicine, Medical University of Gdansk, Poland

**Table S1**. Clinico-pathomorphological characteristic of melanoma patients.

| **Parameter** | **Melanoma patients** |
| --- | --- |
| **Number of cases** | 17 |
| **Sex** (female/male) | 8/9 |
| **Age** (mean [range]) | 63.9 (35-90) |
| **Breslow** (number of patients) |  |
| *≤1.0mm* | 2 |
| *1.0-2.0mm* | 2 |
| *2.1-3.0mm* | 2 |
| *3.1-4.0mm* | 1 |
| *>4.0mm* | 10 |
| **Clark level** (number of patients) |  |
| *I* | 0 |
| *II* | 2 |
| *III* | 6 |
| *IV* | 4 |
| *V* | 5 |
| **Histological type** |  |
| *SSM* | 7 |
| *NMM* | 10 |
| **Non-metastasizing/Metastasizing** (number of patients) | 5/12 |

**Table S2.** Characteristic of melanoma patients and control group included in the TNF-α study.

| **Variable** | **Melanoma patients (n=32)** | **Control group (n=20)** |
| --- | --- | --- |
| **Age** (mean; median; range [years]) | 66; 63; 40-83 | 53; 47; 23-94 |
| *<30* | 0 | 1 |
| *30-39* | 0 | 2 |
| *40-49* | 6 | 8 |
| *50-59* | 5 | 2 |
| *60-69* | 10 | 4 |
| *≥70* | 11 | 3 |
| **Sex** (female/male) | 16/16 | 12/8 |
| **TNF α**  *mean*  *median*  *range [pg/ml]* | 22.80  18.23  3.90-88.00 | 33.83  12.89  3.90-33.83 |
| **Treatment** (Radiotherapy after surgery/Surgery) | 32/0 | 0/20 |

**Table S3.** List of TaqMan probes used and purchased from Thermo Fisher Scientific / Invitrogen.

| **Gene** | **ID:** |
| --- | --- |
| RIPK4 | Hs01062501_m1 |
| BIRC3 | Hs00985030_g1 |
| MAP2K6 | Hs00992389_m1 |
| TRAF1 | Hs01090170_m1 |
| TNFRSF9 | Hs00155512_m1 |
| IL-8 | Hs00174103_m1 |
| IL-6 | Hs00174131_m1 |
| YBX3 | Hs01124964_m1 |
| ROR1 | Hs00938677_m1 |
| PMEL | Hs00173854_m1 |
| RGS2 | Hs01009070_g1 |
| ELK-1 | Hs00901847_m1 |
| HAS3 | Hs00193436_m1 |
| NOTCH3 | Hs01128537_m1 |
| ADAM12 | Hs01106101_m1 |
| TONSL | Hs00273774_m1 |
| TLR3 | Hs01551079_g1 |
| TRIL | Hs04188203_s1 |
| GAPDH | Cat. No. 4326317E |

**Table S4.** Antibodies used for Western Blot, IF and IHC analysis

| **Method** | **Antibody** | **Source** | **Dilution** | **Cat. No.** | **Company** |
| --- | --- | --- | --- | --- | --- |
| Western blot | anti-RIPK4 | rabbit | 1:2000 | 12636 | Cell Signaling Technology |
|  | anti-GAPDH |  |  | 5174 |  |
|  | anti-p38 |  |  | 9212 |  |
|  | anti-phospho-p38 (Thr180/Tyr182) |  |  | 4511 |  |
|  | anti-BIRC3 (c-IAP2) |  |  | 3130 |  |
|  | anti-p65 NF-κB subunit |  |  |  |  |
|  | anti-phospho-p65 NF-κB subunit (Ser636) |  |  |  |  |
|  | anti-MAP2K6 |  |  | SAB4502413 | Sigma -Aldrich/Merck |
|  | HRP-conjugated goat anti-mouse | goat | 1:4000 | 554002 | BD Pharmingen |
|  | HRP-conjugated goat anti-rabbit |  | 1:2000 | 7074 | Cell Signaling Technology |
| IF | anti-p65 NF-κB subunit | rabbit | 1:150 | sc-109 | Santa Cruz Biotechnology |
|  | anti-AlexaFluor 488-conjugated secondary (IgG (H+L) Cross-Adsorbed) | goat | 1:500 | A-11008 | Invitrogen |
| IHC | anti-RIPK4 | rabbit | 1:70 | A8495 | Abclonal |
|  | ImmPRESS HRP REAGENT KIT anti-mouse IgG | horse | ready-to-use | MP-7402 | Vector Laboratories |

**Table S5.** Analysis of gene expression within the JAK-STAT signaling pathway in WM266.4 cells with reduced RIPK4 levels

| Gen | Log2 R/N | p value |
| --- | --- | --- |
| IL-6 | 1.18 | × |
| CSF3R | 0.77 | 0.09 |
| SOCS3 | 0.5 | 0.94 |
| IL7R | 0.36 | × |
| PIM1 | 0.33 | 0.95 |
| OSMR | 0.33 | 0.74 |
| CSF2 | 0.14 | × |
| GHR | 0.08 | 0.99 |
| LIF | 0.02 | 0.99 |
| JAK1 | -0.04 | 0.99 |
| AKT3 | -0.53 | 0.24 |

Log2 R/K - logarithm at base 2 of the ratio of gene transcript levels in siRNA-RIPK4-transfected cells (R) to gene transcript levels in control cells -neg.si (N); p-value - test probability corrected for FDR (false discovery rate); × indicates p-values filtered out in independent filtering during data analysis.
